# Advancing Human Placental Modeling Through Stem-Cell-Derived Trophoblast Organoids and Reprogramming Innovations

**DOI:** 10.3390/biomedicines14081729

**Published:** 2026-07-31

**Authors:** Sukanta Jash, John M. Sedivy

**Affiliations:** Center on the Biology of Aging, Department of Molecular Biology, Cell Biology, and Biochemistry, Brown University, 225 Dyer Street, Providence, RI 02903, USA

**Keywords:** human trophoblast stem cells (hTSCs), induced trophoblast stem cells (iTSCs), pluripotency-independent reprogramming, human pluripotent stem cells (hPSCs), trophoblast lineage specification, trophoblast organoids, placenta-on-a-chip/organ-on-chip models, syncytiotrophoblast and extravillous trophoblast, BMP4–GATA3–TFAP2C signaling axis, maternal–fetal interface and pregnancy disorders

## Abstract

The human placenta is a temporary organ structured to optimize exchange between the maternal and fetal circulatory systems. Its fetal component consists of highly branched chorionic villi, which are anchored to the maternal uterine wall and project into the intervillous space. The outer surface of these villi is lined by a multinucleated, continuous layer called the syncytiotrophoblast, which is supported by an underlying layer of proliferative cytotrophoblast cells and the invasive extravillous trophoblast (EVT). This cellular bilayer forms a selective barrier that directly bathes in maternal blood, allowing for the efficient transfer of oxygen and nutrients while structurally preventing the direct mixing of maternal and fetal blood cells. Human placental studies have been stymied by ethical and accessibility constraints. Stem cell biology has now revolutionized the capacity to model human placental development, in particular with the derivation of human trophoblast stem cells (hTSCs) and organoids. Authentic, self-renewing human trophoblast stem cells (hTSCs) were first derived not from pluripotent stem cells but from primary tissue—first-trimester villous cytotrophoblasts and blastocysts. Derivation from human pluripotent stem cells (PSCs) followed only subsequently, along two principal routes: conversion of naive PSCs, which retain extraembryonic competence, and induction from primed PSCs, as well as by direct reprogramming of somatic cells to induced hTSCs. An important advance underlying these improvements is the mapping of a global reprogramming roadmap. Multi-omic and lineage-tracing experiments have mapped the stepwise transcriptional and epigenetic conversions of fibroblasts to hTSCs, including sequential chromatin reconfiguration, trophoblast gene network activation, and repression of somatic signatures. These results identify major regulatory bottlenecks and intermediate states, improving reprogramming fidelity. The derivation of stem-cell-based trophoblast organoids now enables complex modeling of placental architecture, function, and disease susceptibility in vitro. These organoids accurately recapitulate placental barrier functions and immunological features, allowing for examinations of maternal–fetal health, pregnancy disorders, and placental infection response to viruses like cytomegalovirus and SARS-CoV-2. Looking ahead, the integration of reprogramming and organoid technologies will propel patient-specific and tailor-made models for personalized diagnostics, drug screening, and mechanism studies. As we unravel the molecular ballet of trophoblast induction, such discoveries have the potential to bridge basic translational gaps in reproductive biology and maternal–fetal medicine.

## 1. Introduction

For most of the modern era of stem cell biology, human trophoblast research lagged behind that of the embryo proper because it lacked a self-renewing in vitro system. Mouse trophoblast stem (TS) cells were established from blastocyst and early post-implantation trophoblast in 1998, sustained by FGF4, and became the reference model for the lineage [1]—yet an equivalent human culture remained elusive for two decades. In its absence, the field relied on imperfect surrogates: choriocarcinoma lines such as BeWo, JEG-3 and JAR, which carry malignant genomes and aberrant differentiation, and primary cytotrophoblasts, which are scarce, short-lived, and highly variable between donors [2]. BMP4 treatment of human embryonic stem cells was introduced as a renewable alternative [3], but the identity of the resulting cells proved contentious: when assessed against a stringent panel—a defined marker set, HLA class I profile, *ELF5* promoter methylation, and *C19MC* miRNA expression—BMP4-treated hESCs satisfy only some criteria and are more accurately termed trophoblast-like rather than bona fide trophoblast [4]. This long-standing gap was closed by Okae et al., who defined culture conditions permitting derivation of authentic, self-renewing human TS cells from villous cytotrophoblast and blastocysts [5], establishing the platform on which the reprogramming and organoid advances reviewed here are built.

The first lineage bifurcation in mammalian preimplantation development separates trophectoderm (TE) from inner cell mass (ICM), determining whether cells contribute to extraembryonic support tissues or the embryo proper [6]. This decision has far-reaching implications for reproductive biology, placental health, and pregnancy-related complications such as miscarriage, preeclampsia (a hypertensive disorder of pregnancy associated with deficient EVT invasion, impaired spiral-artery remodeling, an anti-angiogenic shift [elevated sFLT-1/PlGF], and endothelial dysfunction), and intrauterine growth restriction [7]. Single-cell transcriptomics and epigenomics analyses indicate that trophoblast cell fate is controlled by continuous chromatin reconfiguration and a stepwise activation of the TE transcriptional regulators (*GATA2*, *GATA3*, *TFAP2A*, *TFAP2C*). In particular, this is coupled with silencing of pluripotency genes, supporting the idea that loss of pluripotency is a prerequisite to TE specification [8].

Human pluripotent stem cells (hPSCs), whether embryonic or induced, have really changed how researchers study developmental mechanisms in the lab. They sidestep a lot of the old limitations in embryology [9]. The capacity to generate trophoblast is not a uniform feature of pluripotency but is state-dependent and reflects defined molecular properties. Human naive PSCs approximate the pre-implantation epiblast: they are globally DNA-hypomethylated and retain a chromatin and transcriptional configuration permissive for extraembryonic fate [10]. Consistent with this, naive—but not primed—human PSCs efficiently give rise to blastocyst-stage trophectoderm and its downstream derivatives (cytotrophoblast, syncytiotrophoblast, and trophoblast stem cells) upon inhibition of ERK/MAPK and Nodal signaling, whereas this competence is progressively lost as cells acquire the primed state, at which point their extraembryonic potential switches toward amnion [11,12,13]. The greater trophoblast competence of naive cells is therefore attributable to a specific pre-implantation-like epigenetic and signaling state rather than to an intrinsic cue of the pluripotent state as such. Differentiation methods have moved well beyond flat 2D cultures; now, scientists use 3D organoids and organ-on-chip systems. Researchers have also managed to generate self-renewing human trophoblast stem cells (hTSCs) from blastocysts, early placenta, and hPSCs. This lets them dive deep into how these cells renew themselves and later become syncytiotrophoblast or extravillous trophoblast [5,12].

Naive hPSCs are a pre-implantation epiblast-like ground state (LIF/MEK-ERK-independent self-renewal, global DNA hypomethylation, two active X chromosomes), distinct from the post-implantation-like primed state captured by conventional hESC/iPSC culture. Naive hPSCs are markedly more competent than primed cells for trophoblast differentiation. This advantage reflects specific features retained in the naive state: an active, hypomethylated C19MC cluster [14], DNA hypomethylation and accessible chromatin at trophoblast regulators, and residual trophectoderm-associated transcriptional priming that resembles blastocyst trophectoderm [14]. These features are progressively erased as cells acquire the primed state, narrowing lineage plasticity across the pre- to post-implantation transition [13,14]. Multi-omic studies back this up: hTSCs from naive hPSCs resemble closely trophoblast cells directly isolated from the blastocyst, even showing post-implantation TE features. Epigenetic regulation stands at the center of this process. Primate-specific microRNA clusters like C19MC are essential for strong trophoblast differentiation, underscoring how crucial epigenetic reprogramming is for lineage commitment. This insight paves the way for engineering hPSCs with specific trophoblast potential—a notable breakthrough for both developmental research and regenerative medicine [15].

## 2. Trophoblast Derivation from Pluripotent Stem Cells (Table 1)

### 2.1. Methodological Advances in Trophoblast Derivation from Pluripotent Stem Cells

**Figure 1:**
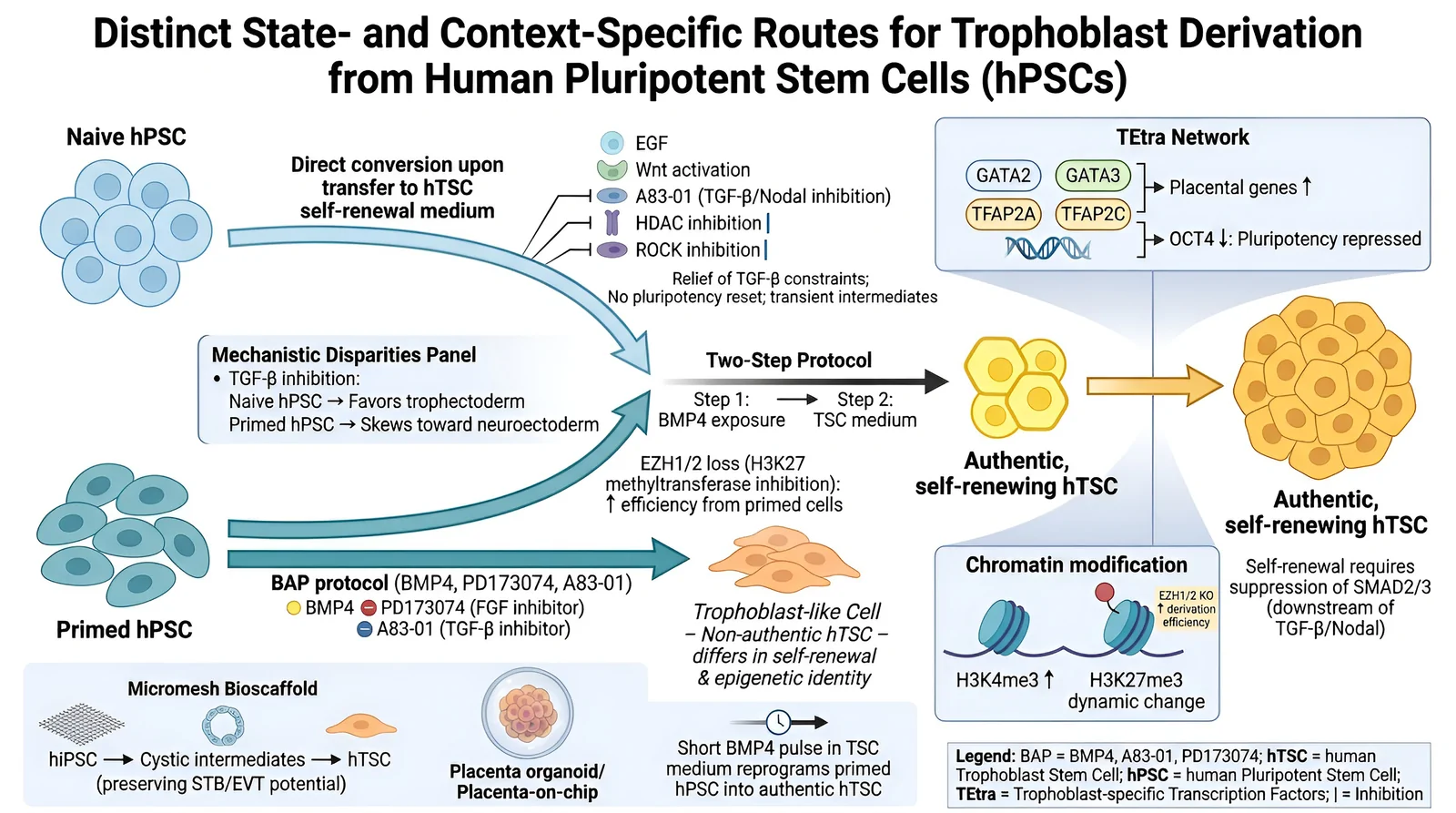
Methodological and molecular complexities in the generation of trophoblast stem cells (hTSCs) from pluripotent stem cells.

The foundation for authentic human trophoblast stem cell (hTSC) culture was laid by Okae et al., who defined the medium conditions that finally permitted long-term self-renewal of human trophoblast in vitro [5]. Working from first-trimester villous cytotrophoblasts—and, equivalently, from blastocysts—they showed that combined activation of Wnt (CHIR99021) and EGF signaling together with inhibition of TGF-β/Nodal (A83-01, SB431542), histone deacetylase (VPA) and ROCK (Y-27632) sustains karyotypically stable, self-renewing hTSCs that retain a cytotrophoblast-like identity (*TP63*, *GATA3*, *TFAP2C*, *ELF5*, *KRT7*), a hypomethylated *ELF5* promoter and *C19MC* expression, and remain bipotent for syncytiotrophoblast and extravillous trophoblast differentiation (Figure 1). Notably, this self-renewal depends on suppression—not activation—of SMAD2/3 signaling, in direct contrast to mouse trophoblast stem cells.

This blastocyst/cytotrophoblast-derived system remains the gold-standard reference model against which all subsequent PSC-derived and reprogramming-based hTSC protocols are benchmarked. Reproducing this hTSC state from pluripotent sources—without access to primary placental or blastocyst material—has since become the central objective of the derivation strategies discussed below, and the fidelity of each is judged by how closely its products match the molecular and functional benchmarks set by Okae-derived hTSCs (Figure 1).

It is now possible to derive human trophoblast stem cells (hTSCs) from human pluripotent stem cells (hPSCs) via state- and context-specific methodologies that elucidate essential characteristics of trophoblast commitment. Naive hPSCs convert to hTSCs upon transfer into hTSC self-renewal medium—EGF with inhibition of TGF-β/Nodal (A83-01) and Wnt activation, alongside HDAC and ROCK inhibition—with relief of TGF-β-dependent constraints on naive self-renewal further facilitating the transition [8]. This conversion proceeds without an intervening pluripotent reset, although lineage-tracing data indicate that cells pass through transient intermediate states rather than switching identity instantaneously [8]. In contrast, primed hPSCs require the BAP protocol—BMP4 together with inhibition of FGF (PD173074) and ACTIVIN/TGF-β (A83-01)—to generate trophoblast-like cells [16]. These cells are useful for modeling early trophoblast emergence but are not equivalent to authentic, self-renewing hTSCs derived from blastocyst or placental tissue, differing in self-renewal capacity and epigenetic identity (Figure 1). A two-step protocol—initial exposure to BMP4 followed by maintenance in trophoblast stem cell medium—effectively produces authentic self-renewing TSCs from both naive and primed states, with epigenetic modulation (including H3K27 methyltransferase loss) further increasing derivation efficiency from primed cells [17]. The mechanistic disparities among pluripotent states are pronounced: TGF inhibition in naive hPSCs fosters trophectoderm formation, whereas in primed cells, it skews towards neuroectoderm, highlighting unique lineage wiring [18].

Simultaneous improvements in culture microenvironments boost efficiency and fidelity even more. For example, micromesh bioscaffold systems move hiPSCs through cystic intermediates to proliferative hTSCs while keeping trophoblast programs and the ability to differentiate into syncytiotrophoblast and extravillous trophoblast [19]. 3D organoid and placenta-on-chip platforms, on the other hand, better mimic the structure and function of living organisms [20]. A short BMP4 treatment in TSC maintenance settings can also change primed hPSCs into trophoblast stem-like cells that look, express genes, and have long-term self-renewal that is similar to real hTSCs [21] (Figure 1).

**Figure 2:**
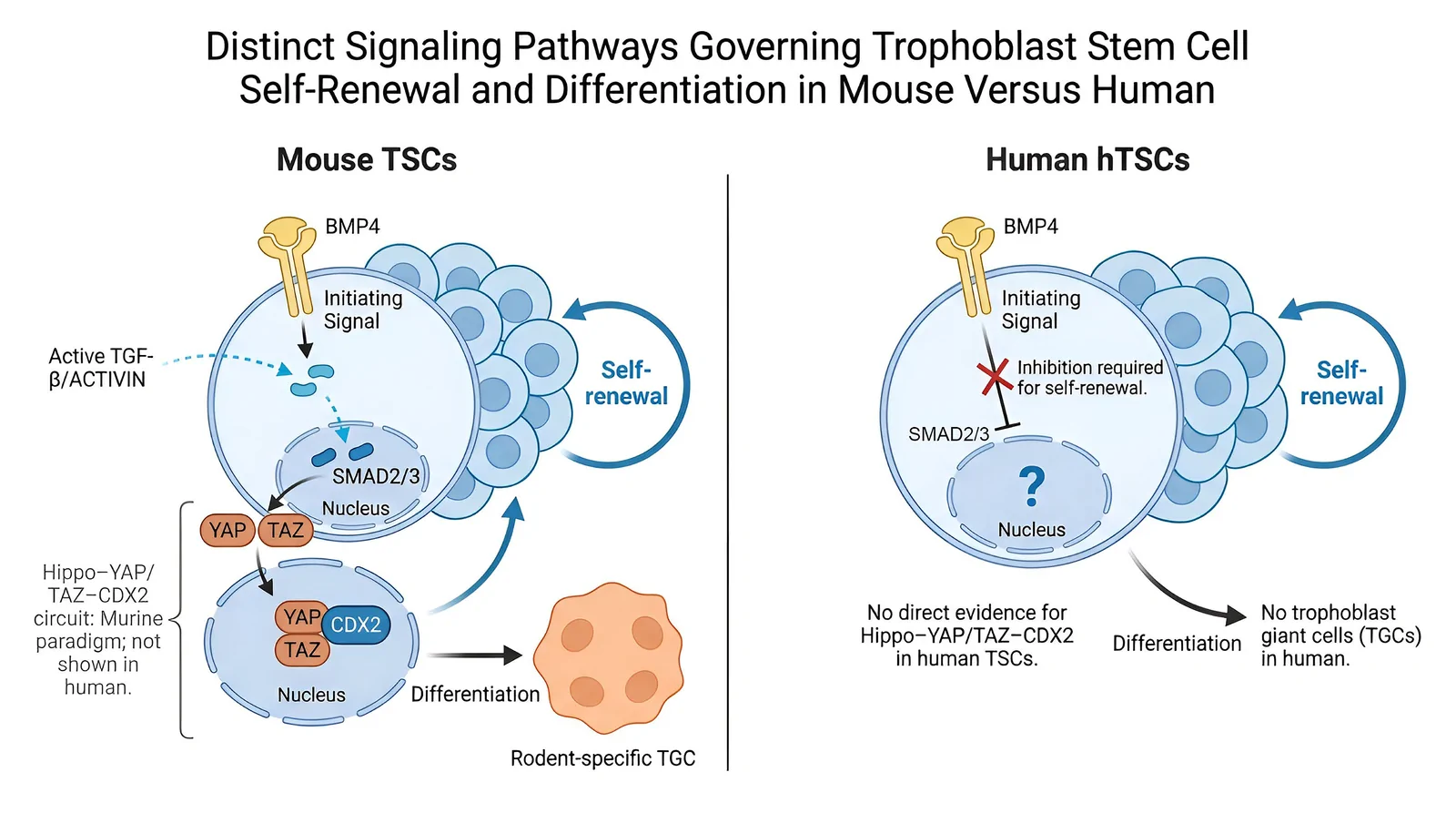
Distinct molecular pathways in the generation of trophoblast stem cells (TSCs) between mouse and human.

The TEtra network (*GATA2*, *GATA3*, *TFAP2A*, *TFAP2C*) concurrently activates trophoblast programs and represses pluripotency by binding to both placental gene and OCT4 regulatory areas [8]. Chromatin profiling reveals synchronized alterations in H3K4me3 and H3K27me3 throughout the transition from hPSC to hTSC, with the deletion of EZH1/2 significantly enhancing hTSC derivation [17]. In the mouse, YAP/TAZ effectors of the Hippo pathway interact with CDX2 to control the balance between trophoblast stem cell self-renewal and differentiation into trophoblast giant cells, linking mechano-signaling to trophoblast fate [22]. Importantly, trophoblast giant cells are a rodent-specific lineage with no direct human counterpart, and this Hippo–YAP/TAZ–CDX2 circuit has not been shown to operate equivalently in human trophoblast; it should therefore be regarded as a murine paradigm awaiting validation in human cells. BMP4 is broadly recognized as a principal initiating signal for trophoblast specification in both mouse and human, though the downstream signaling requirements for maintaining that identity diverge sharply between species [23] (Figure 2).

**Table 1:** Comparison of Trophoblast Derivation Methods.

Derivation Method	Source Cell Type	Key Signaling Components	Culture Duration	Resulting Cell State	References
Direct outgrowth in “TS medium” on collagen IV	First-trimester villous cyto trophoblast (CTB)	Wnt activation (CHIR99021) + EGF; inhibition of TGF-β/Nodal (A83-01 + SB431542), HDAC (VPA), ROCK (Y-27632)	Long-term self-renewing; >30 passages/~5 months, karyotypically stable	Self-renewing hTSCs (CTB-like): TP63^+^, GATA3^+^, *TFAP2*C^+^, *TEAD4^+^*, *ELF5^+^*, *KRT7^+^*; hypomethylated *ELF5*; *C19MC^+^*; bipotent (→STB, EVT)	[5]
Blastocyst outgrowth in the same “TS medium”	Blastocyst (trophectoderm-derived)	Identical to above	Equivalent long-term self-renewal	hTSC lines transcriptionally/functionally equivalent to CTB-derived hTSCs	[5]
Direct differentiation	Naive hPSCs	Moderate BMP4 ± minimal inhibitors	5–7 days	Self-renewing hTSCs with postimplantation cytotrophoblast-like identity Note: No current model recapitulates preimplantation trophectoderm. Trophoblast-like or amnion-like population (disputed identity) resembling first-trimester syncytiotrophoblast	[12]
BMP4-based protocol	Primed hPSCs	BMP4 + A83-01 + PD173074 (BAP)	8 days	Mixed trophoblast population resembling first-trimester syncytiotrophoblast	[3,24]
Two-step BMP4 + TSCM	Primed hPSCs	BMP4 (step 1) + TSCM (step 2)	3–5 days (step 1) + continuous	Trophoblast stem-like self-renewing hTSCs with enhanced efficiency	[17]
Micromesh bioscaffold	hiPSCs	Culture-dependent signaling	10–14 days	Proliferative hTSCs from cystic intermediates with full differentiation potential	[19]
3D organoid/placenta-on-chip	hiPSC-derived trophoblasts	Perfusion + matrix interactions	7–21 days	Polarized, invasive syncytial structures with physiological functionality	[20]
TSCM short-term treatment	Primed hPSCs	BMP4 in TSCM	3–5 days	Trophoblast stem-like cells (TSLCs) equivalent to bona fide hTSCs	[21]
PRC2-inhibited protocol	Primed hPSCs	Inhibition of H3K27me3 deposition	Variable	Enhanced trophectoderm commitment with restricted mesoderm potential	[17]

### 2.2. Direct Reprogramming to Induced Trophoblast Stem Cells

**Figure 3:**
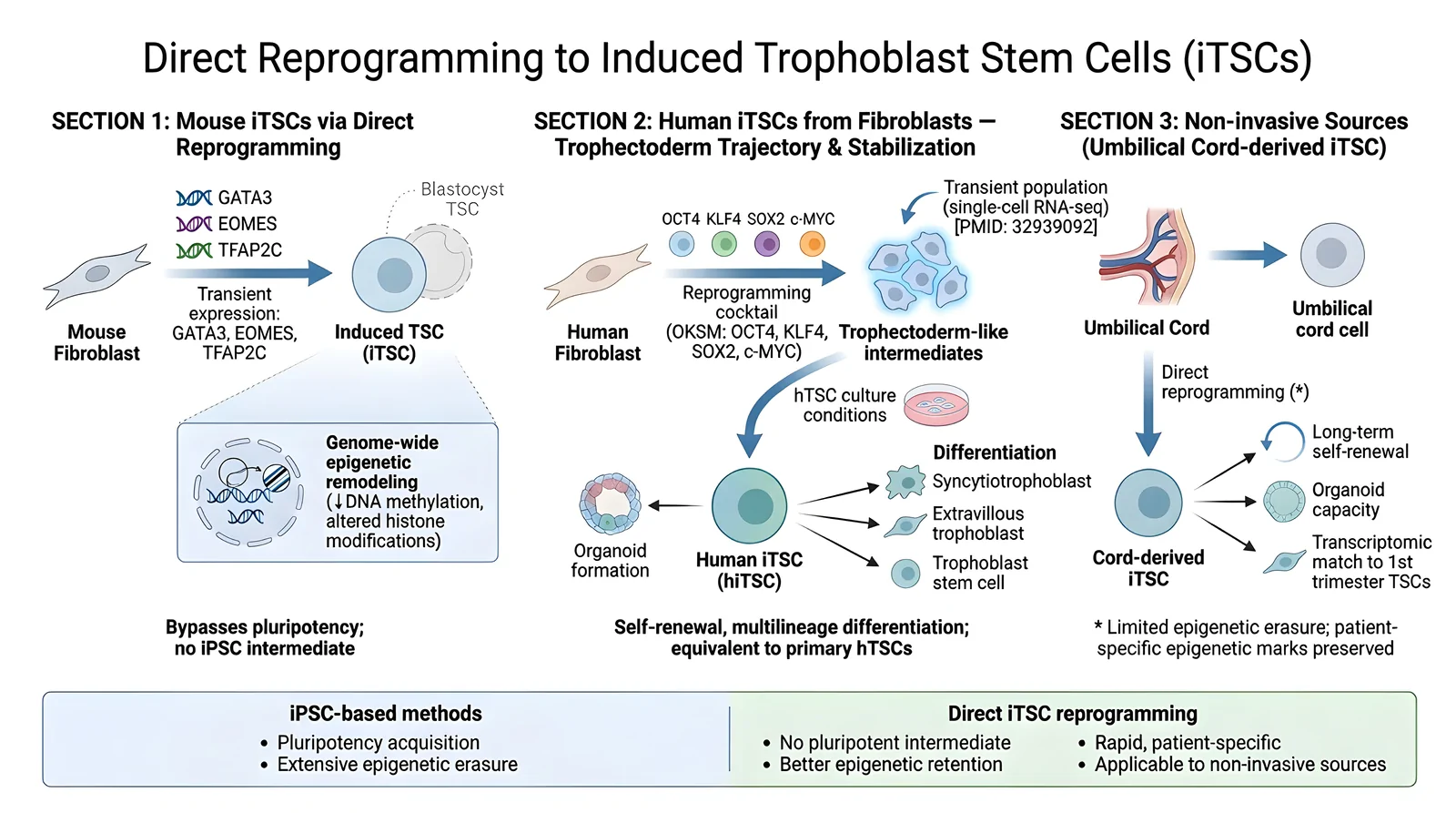
Direct reprogramming roadmap for Trophoblast stem cells.

A transformative development has been the establishment of direct reprogramming approaches that bypass pluripotency entirely (Figure 3). Benchetrit and colleagues demonstrated that transient expression of three transcription factors—*GATA3*, *EOMES*, and *TFAP2C*—in mouse fibroblasts induces stable induced trophoblast stem-like cells (iTSCs) with transcriptional, epigenetic, and functional properties closely matching blastocyst-derived TSCs [25]. Importantly, these mouse iTSCs underwent extensive transcriptional and functional reprogramming to a trophoblast stem cell-like state without evidence of passage through a pluripotent intermediate (Figure 3). Genome-wide DNA methylation and histone modifications were not, however, directly profiled in these cells; the extent of epigenetic reprogramming—and whether it parallels that of induced pluripotent stem cells—therefore remains to be established, as does whether the approach is achievable in human cells.

In humans, Liu and colleagues mapped the reprogramming trajectories of fibroblasts to both naive and primed pluripotent states using single-cell transcriptomics, revealing an unexpected role for trophectoderm-associated gene expression during the reprogramming process [26]. This transient trophoblast-like subpopulation could be captured and stabilized using hTSC culture conditions, enabling direct derivation of induced trophoblast stem cells from somatic cells (Figure 3). The resulting iTSCs were functionally equivalent to primary hTSCs, demonstrating self-renewal capacity and multilineage differentiation potential. Subsequently, optimized protocols employing the OKSM (*OCT4*, *KLF4*, *SOX2*, *c-MYC*) reprogramming factors combined with selective culture conditions have enabled efficient generation of patient-specific iTSCs from dermal fibroblasts [27]. This approach offers significant advantages for disease modeling, as cells can be derived from readily accessible adult tissues without requiring embryonic or first-trimester placental material.

Recent work has extended direct reprogramming to term tissues, generating bona fide iTSCs from umbilical cord cells collected non-invasively at birth [28,29]. These iTSCs fulfilled criteria for bona fide first-trimester trophoblasts, including long-term self-renewal, differentiation into hormone-producing syncytiotrophoblast and invasive extravillous trophoblast, organoid-forming capacity, and transcriptomic similarity to primary first-trimester hTSCs. Notably, this approach may better preserve patient-specific epigenetic marks compared to conventional iPSC-based methods, as cells undergo limited reprogramming without extensive epigenetic erasure associated with naive pluripotency acquisition.

## 3. Transcriptional and Epigenetic Networks in Trophoblast Cell Fate Determination

### 3.1. Epigenetic and Transcriptional Regulation of Trophoblast Lineage Specification

**Figure 4:**
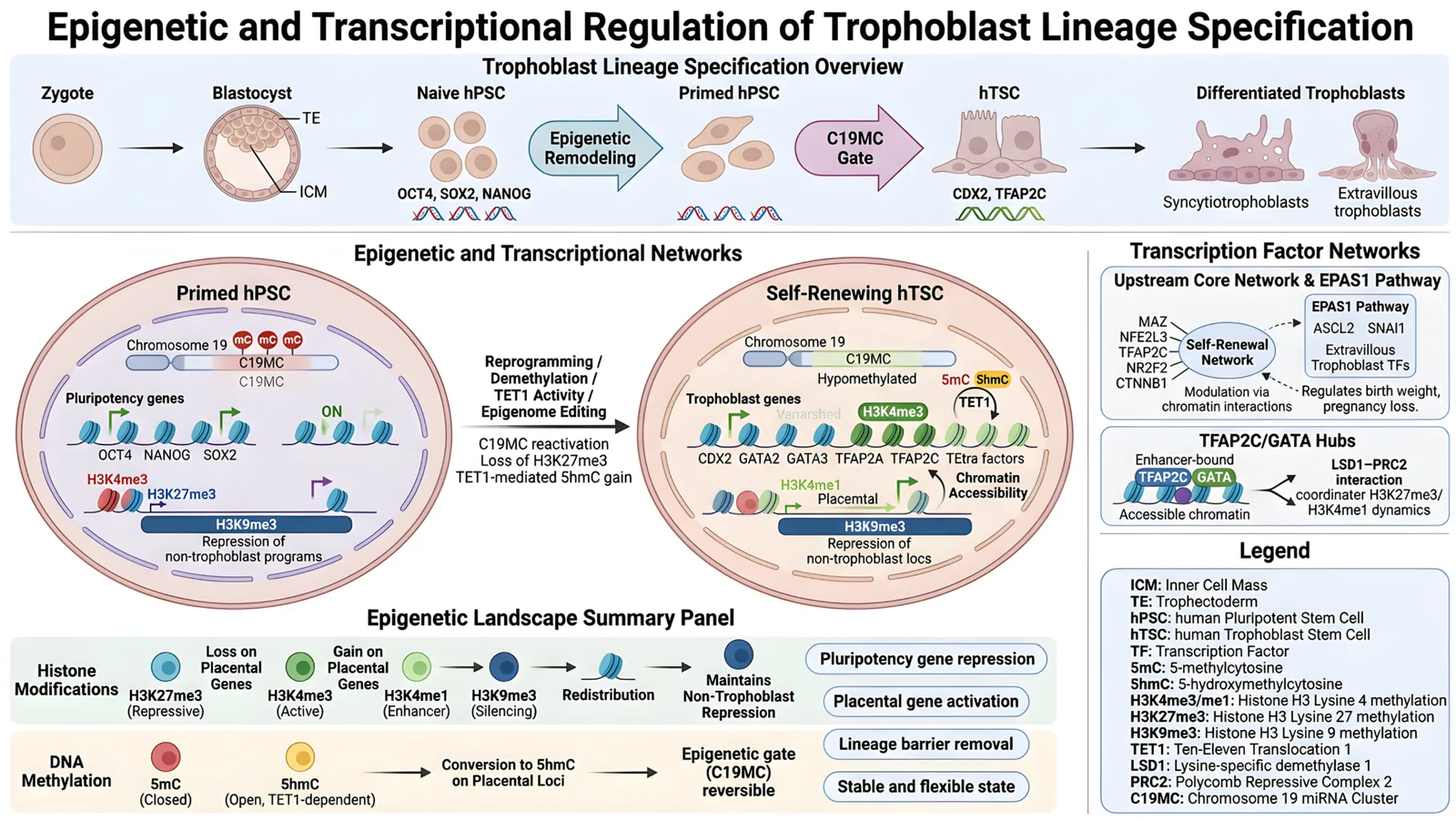
Molecular pathways regulating trophoblast stem cell (hTSC) lineage specification.

Trophoblast lineage commitment is governed by significant epigenetic remodeling that differentiates self-renewing trophoblast stem cells (hTSCs) from pluripotent precursors (Figure 4). Single-cell atlases from zygote to mid-gestation delineate a fundamental self-renewal network—including *MAZ*, *NFE2L3*, *TFAP2C*, *NR2F2*, and *CTNNB1*—validated by siRNA knockdown as critical for hTSC maintenance [30]. The integration of chromatin accessibility, three-dimensional chromatin interactions, and transcriptomics suggests that EPAS1 functions as an upstream regulator of extravillous trophoblast transcription factors (Figure 4), including *ASCL2* and *SNAI1*, hence associating this pathway with variations in birth weight and pregnancy loss [31].

The chromosome 19 microRNA cluster C19MC offers a primate-specific regulatory layer (Figure 4), functioning in naive human embryonic stem cells (ESCs) while being epigenetically suppressed in primed cells through DNA methylation and chromatin remodeling [14]. Reactivating C19MC with targeted genome/epigenome editing reinstates trophoblast competence in otherwise resistant primed hPSCs, illustrating that this cluster operates as a reversible molecular gate to the trophoblast lineage [14].

Histone modifications and DNA methylation reshape chromatin as cells specialize. They silence pluripotency genes and switch on regions that drive placental development. In these early stages, bivalent regions marked by both H3K27me3 and H3K4me3 start to change (Figure 4). Cells lose H3K27me3, and the H3K4me1 pattern shifts, kicking off trophoblast-specific gene programs and blocking other cell fates [17]. TET1 refines this whole process. In human trophoblast stem cells (hTSCs), TET1 sticks to regions that overlap with active histone marks and TFAP2C-bound enhancers. In mouse trophoblast, the balance between 5mC and 5hmC distinguishes stem from differentiated cells and highlights candidate regulators of the trophoblast stem cell state [32]. An equivalent 5mC/5hmC analysis has not yet been performed in human trophoblast, and given the species divergence in trophoblast epigenetic regulation, this should be treated as a murine observation requiring human confirmation. When chromatin accessibility, histone modifications, DNA methylation, and hydroxymethylation work together, the result is an epigenetic landscape that is both stable and flexible (Figure 4). This dynamic framework maintains trophoblast cell growth and self-renewal, while permitting appropriate differentiation [14,30,31,32].

### 3.2. Key Epigenetic Modifications in Trophoblast Lineage Specification

#### 3.2.1. H3K4me3, and H3K4me1

Numerous interconnected epigenetic processes regulate the commitment and maintenance of trophoblast lineage (Figure 4). Repressive H3K27me3, deposited by PRC2, silences pluripotency and developmental regulators; the loss of EZH1/2 depletes H3K27me3 and significantly enhances the derivation of hTSCs from primed hPSCs, underscoring this mark as a barrier to trophoblast specification, particularly at bivalent loci transitioning to active or stably repressed states [17]. Active marks H3K4me3 and H3K4me1 redistribute across promoters and enhancers of placental genes and TEtra factors (*GATA2*, *GATA3*, *TFAP2A*, TFAP2C), with H3K27me3–H3K4me1 state transitions, partly mediated by LSD1–PRC2 interactions, orchestrating trophoblast-specific expression patterns [8,17].

#### 3.2.2. DNA Methylation (5-Methylcytosine), 5-Hydroxymethylcytosine (5hmC), and C19MC (Chromosome 19 MicroRNA Cluster)

DNA methylation and 5-hydroxymethylcytosine (5hmC) further refine lineage potential. The primate-specific *C19MC* cluster is hypomethylated and active in naive hPSCs but hypermethylated and muted in primed cells; targeted reactivation restores trophoblast competence, suggesting that *C19MC* functions as a reversible epigenetic gate to the trophoblast lineage [14]. In the same mouse study [32], *TET1*-mediated oxidation of 5mC to 5hmC at trophoblast enhancers and promoters is associated with active transcription and distinguishes self-renewing trophoblast stem cells from their differentiated progeny, with *TET1*-occupied, *TFAP2C*-enriched regions acting as candidate regulatory elements. As above, these findings derive from mouse trophoblast and have not been demonstrated in human trophoblast stem cells (Figure 4).

#### 3.2.3. Chromatin Accessibility and H3K9me3

Concurrently, chromatin accessibility analysis reveals that hTSCs consolidate their trophoblast identity by gaining open chromatin at placental regulators such as *CDX2* and *TEtra* factors and closing *OCT4*, *NANOG*, and *SOX2* [30]. H3K9me3-enriched heterochromatin further represses non-trophoblast programs, hence preserving lineage fidelity. These coordinated histone marks, DNA methylation/hydroxymethylation states, *C19MC* activity, and accessibility landscapes create a stable but adjustable epigenetic framework that allows trophoblast specification and limits other possibilities (Figure 4).

## 4. Disease Modeling Using Reprogrammed Trophoblast Lineage Cells (Table 2)

### 4.1. Disease Modeling and Clinical Applications of Reprogrammed Trophoblast Lineage Cells

**Figure 5:**
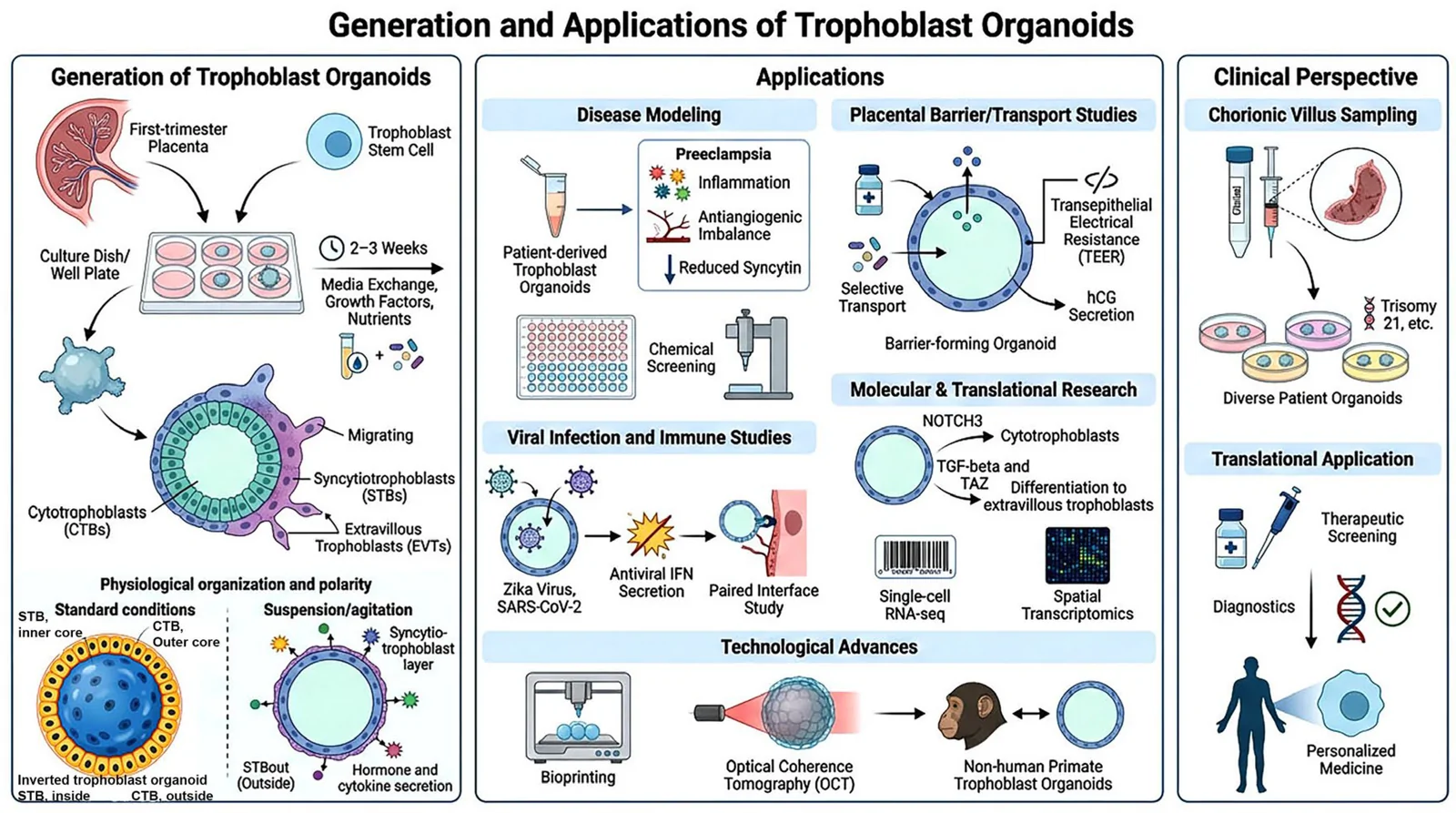
Trophoblast organoids and their applications.

The use of patient-derived induced pluripotent stem cells (iPSCs) presents a novel opportunity to model pregnancy problems at the trophoblast level (Figure 5). The pathogenesis of preeclampsia is replicated in trophoblasts that lack proper syncytialization and have a reduced ability to respond to hypoxia, suggesting that chromatin and post-transcriptional regulation play a role in this disease [33]. MA et al. (2019) [34] and Zhang et al. (2023) [35] found that adrenomedullin (ADM), a placental peptide with lower levels in at-risk pregnancies during the first trimester, is a critical regulator of trophoblast differentiation and a potential biomarker and treatment for early-onset preeclampsia.

Gestational trophoblastic disease, encompassing *NLRP7*-mutant moles, has been effectively modeled using patient-derived iPSC trophoblasts, demonstrating that *NLRP7* dysfunction leads to premature pluripotency loss and BMP4-dependent differentiation; BMP inhibition rectified these phenotypes, suggesting potential therapeutic approaches [36]. Single-cell RNA sequencing of preeclamptic placentas reveals unique trophoblast subtypes exhibiting dysregulated adhesion, migration, and immune tolerance pathways (Figure 5). Additionally, NOTCH3 signaling is identified as a pivotal regulator of human trophoblast stem cell expansion and differentiation, with its dysregulation associated with placental pathology [37,38].

Derivation of disease-specific human stem cells (hTSCs) from patient-induced pluripotent stem cells (iPSCs) offers genetically tractable, temporally controllable, and ethically suitable disease modeling for therapeutic screening (Figure 5) [9]. Recent developments in monolayer cultures, 3D organoids, and placenta-on-chip technologies improve structural fidelity and the recreation of the maternal–fetal interface [39]. Reprogramming mature placental cytotrophoblasts into induced trophoblast stem-like cells reinstates developmental plasticity, facilitating the investigation of early gestational events that influence subsequent placental problems such as preeclampsia and preterm birth [28]. Integration with multi-omics and weighted correlation network analyses makes it possible to conduct complete mechanistic dissections and identify therapeutic targets that go beyond single-gene techniques (Figure 5).

**Table 2:** Disease Models Created Using Reprogrammed Trophoblast Lineage Cells (Figure 5).

Disease/Condition	Source Material	Key Findings	Therapeutic Insights	References
**Preeclampsia (PE)**	Patient-derived iPSCs from placental tissue	Defective syncytialization, blunted hypoxic response, dysregulation of cell adhesion and migration modules (*CTL9*, *EOPE1*, *EOPE2*)	Identification of gene modules controlling invasion and oxygen response; potential targets for therapeutic intervention without marked DNA methylation changes; consideration of regulatory mechanisms beyond epigenetic modifications	[33]
**Early-Onset Preeclampsia (EOPE)**	iPSCs derived from umbilical cord cells of affected pregnancies	Reduced trophoblast invasive capacity under hyperoxic conditions; dysregulation of oxygen response mechanisms; aberrant weighted correlation network modules	Targeting dysregulated gene networks controlling invasiveness; modulation of oxygen-sensing pathways; potential role of adrenomedullin signaling; candidate therapeutic targets validated through in vitro disease models	[34,35]
**Gestational Trophoblastic Disease (Hydatidiform Moles)**	Patient-derived iPSCs harboring NLRP7 mutations	Precocious pluripotency downregulation, premature differentiation marker activation, excessive syncytiotrophoblast maturation, BMP4-dependent phenotypes	BMP4 pathway inhibition corrects phenotypes; identifies NLRP7 as essential regulator of developmental fate decisions; demonstrates pharmacological rescue potential; validates iPSC models for genetic placental disease	[36]
**Placental Insufficiency (General)**	Multiple patient-derived iPSC lines with various genetic backgrounds	Cellular and molecular defects in trophoblast commitment, expansion, and differentiation; tissue-specific gene dysregulation; altered chromatin accessibility at trophoblast regulatory regions	Systematic interrogation of genetic perturbations affecting placental development; compound screening for therapeutic targets; functional genomic approaches enabling discovery of novel regulators	[40]
**NOTCH3-Associated Placental Dysfunction**	iPSC-derived trophoblast stem cells from affected pregnancies	Impaired progenitor expansion, premature syncytial differentiation, reduced stemness marker expression when NOTCH3 signaling disrupted	NOTCH3 signaling pathway as therapeutic target; restoration of canonical NOTCH3 signaling to promote self-renewal and normal differentiation balance	[37]

By integrating advanced differentiation protocols with iPSC reprogramming technology, these disease models demonstrate the ability to generate physiologically relevant in vitro systems that replicate pregnancy pathology (Figure 5). The development of new biomarkers for early prediction and therapeutic targets for intervention in pregnancy complications that do not yet have preventative or curative treatments beyond delivery can be aided by these systems, which allow for the systematic characterization of disease mechanisms at the cellular and molecular level.

### 4.2. Current Limitations and Future Directions in Trophoblast Lineage Specification

Research into in vitro-derived trophoblasts highlights a complex developmental landscape where identity and plasticity are paramount. Transcriptomic evidence confirms that BMP4-induced trophoblasts from primed hPSCs mirror authentic placental characteristics and villous cells [24], effectively resolving earlier debates regarding their similarity to amniotic tissue. However, recent findings reveal unexpected diversity: pluripotent cells can generate two distinct trophectoderm lineage stem cells. These include a CDX2-negative population similar to primary hTSCs and a CDX2-positive putative trophectoderm stem cell (hTESC), both possessing unique transcriptional profiles and differentiation requirements [41]. This variety suggests that current methodologies only capture a fraction of trophoblast states, impacting our ability to engineer specific subtypes.

While human naive epiblast cells typically lose their extraembryonic lineage potential upon transitioning to primed pluripotency [11], targeted interventions show this can be reversed. In the mouse, for example, modulating *Sox2* expression allows naive pluripotent cells to differentiate into both embryonic germ layers and extraembryonic trophoblast without compromising other developmental potentials [42]. This expanded lineage potential has been shown in murine cells and has not been established for human naive PSCs, in which trophoblast competence is governed by partly distinct determinants. This demonstrates that the pluripotent state is a dynamic landscape where epigenetic manipulation can fine-tune cell fate.

To advance the field, integrating single-cell multiomics, 3D culture, and computational modeling is essential. These technologies can identify transitional cell states and establish molecular links between signaling and epigenetics [43]. By applying machine learning to multiomic data, researchers can move from descriptive cataloging to the rational engineering of trophoblasts with specified functional properties, ultimately driving clinical translations in reproductive medicine.

## 5. Stem-Cell-Derived Trophoblast Organoids: Advanced Platforms for Modeling Placental Pathologies and Enabling High-Throughput Therapeutic Screening

Recent advancements in stem cell biology have facilitated the creation of three-dimensional trophoblast organoids derived from pluripotent and trophoblast stem cells, offering physiologically appropriate models for studying placental development and associated pathologies (Figure 5). These organoids mirror essential architectural and cellular characteristics of in vivo tissue, presenting revolutionary prospects for mechanistic exploration and therapeutic advancement [9,44,45].

### 5.1. Development and Characterization of Trophoblast Stem Cell-Derived Organoids

Human trophoblast stem cells are a key resource for building organoids. Lately, researchers have figured out that tweaking the culture environment and signaling molecules lets them guide these cells to form organoids that really match what happens in the body (Figure 5). These human trophoblast stem cells, whether derived from placental tissue, blastocysts, or reprogrammed iPSCs, self-renew and differentiate into multiple trophoblast lineages, providing the source material for organoid formation. Haider et al. and Turco et al. independently generated the first self-renewing human trophoblast organoids in 2018 [40,46,47]; both models display an inverted architecture, with the syncytiotrophoblast facing inward rather than forming the outer surface as it does in vivo. Better culture conditions help these cells form organoids with syncytiotrophoblast layers that act as real, functional barriers [44]. On top of that, single-cell transcriptomics has identified NOTCH3 as a major regulator of trophoblast differentiation [37]. This self-renewal network—*MAZ*, *NFE2L3*, *TFAP2C*, *NR2F2*, and *CTNNB1*—was defined in single-cell placental atlas and hTSC data (see above) [30] but has not yet been functionally interrogated in trophoblast organoids specifically; extending this analysis to the organoid setting remains an open direction for the field.

### 5.2. Molecular Regulation and Signaling Pathways

Lately, single-cell studies and transcriptome analysis on trophoblast organoids have uncovered several fascinating regulatory networks behind how trophoblasts develop and function. The NOTCH3 signaling pathway plays a critical role in regulating the proliferation and differentiation of the human placenta (Figure 5). Expression of NOTCH3 is primarily localized to proliferating cytotrophoblast progenitors. Consequently, inhibition of this pathway promotes differentiation into syncytiotrophoblasts [37]. Then, there is transforming growth factor-beta signaling, which steers how extravillous trophoblasts in organoids mature. This pathway matters for proper invasion of trophoblasts and the right decidualization response [48]. Another player is the transcription factor *TAZ*, which really shapes extravillous trophoblast development by boosting genes linked to EVT identity, cell movement, and survival [49] (Figure 5).

### 5.3. Organoid Architecture and Barrier Function

A significant breakthrough in the technology of trophoblast organoids has been the generation of physiological polarity that mirrors the natural placenta structure, which has an outer layer of syncytiotrophoblast (STB) [50]. Static culture conditions result in inverted polarity with internalized STBs; however, when grown in suspension and gently agitated, the organoids spontaneously rotate and restore physiological polarity with correct apical-basal orientation (Figure 5). This results in increased secretion of STB-associated hormones, such as human chorionic gonadotropin (hCG) and interferon-α2, and the formation of large syncytia with more than 50 nuclei. Following these developments, column-type barrier systems have been generated from trophoblast organoids that show continuous syndecan-1-positive syncytiotrophoblast coverage and maturation similar to primary trophoblasts [44]. These systems allow the quantitative measurement of permeability coefficients and the transfer of small molecules, providing a scalable and physiologically relevant system for the study of placental transport, drug safety, and therapy during pregnancy (Figure 5).

## 6. Disease Modeling Applications

Trophoblast organoids are highly useful tools for research into various diseases, particularly those involving placental abnormalities. Research into infections has shown that Zika virus has a detrimental effect on the structure of the organoid and the process of syncytialization [51]. Single-cell data have shown that there is a lack of hTSC stemness and cytotrophoblast proliferation, characteristics also seen in preeclampsia. Human cytomegalovirus (HCMV) infection, although non-productive in human trophoblast stem cells and their derivatives, dysregulates genes governing cell identity, differentiation, and Wnt signaling, suggesting an indirect mechanism by which infection could perturb placental function. Research into SARS-CoV-2 infection has shown that *ORF3a* has an effect on the process of maturation of the syncytiotrophoblast and invasiveness, altering autophagy, tight junctions, and vesicle secretion [52,53]. These studies show the utility of organoids in understanding viral-induced teratogenesis and maladaptation of the placenta (Figure 5).

### 6.1. Preeclampsia and Placental Insufficiency Modeling

Preeclampsia, the most common hypertensive disorder of pregnancy, is associated with defective trophoblast invasion, abnormal spiral artery remodeling, and endothelial dysfunction (Figure 5). Recent breakthroughs in preeclampsia modeling using a combination of stem cells and organoids, coculture, and microphysiological systems have greatly advanced our current understanding of the underlying molecular mechanisms of preeclampsia. Preeclampsia-modeling organoids—generated by exposing trophoblast organoids to disease-relevant stressors such as hypoxia—express dysregulated genes associated with inflammation, angiogenesis, and trophoblast differentiation [54,55]. It should be noted that, because organoids can only be established from first-trimester cytotrophoblast or blastocyst tissue, the eventual clinical trajectory of the donor pregnancy is generally unknown at the time of derivation; consequently, most “preeclampsia organoids” model the disease through such stressors rather than through tissue with a prospectively confirmed diagnosis, an important caveat for causal interpretation of these studies. The use of organoids to test stem cell-derived EVs in preeclampsia modeling has also demonstrated promising therapeutic potential, with placental EVs promoting trophoblast migration through CD147 signaling, a transmembrane glycoprotein that induces matrix metalloproteinases and supports trophoblast motility and invasion [56], and umbilical cord-derived MSC exosomes carrying *miR 146a 5p* alleviating hypoxia-induced apoptosis through inhibition of TRAF6/NF κB signaling [57], underscoring organoids’ translational value in preeclampsia research. It should be emphasized, however, that current organoid-based disease modeling studies remain largely descriptive—identifying dysregulated genes, pathways, and candidate biomarkers—and have not yet been shown to be predictive of patient prognosis or to directly inform therapeutic decision-making.

### 6.2. High-Throughput Therapeutic Screening Applications

With trophoblast organoids and HTS, a new era is now unfolding in drug discovery for obstetrics. Because organoids better preserve tissue architecture and cell–cell interactions than 2D culture, they are hypothesized to more faithfully predict in vivo drug responses [44,58,59]; however, this concordance has not been directly validated, in part because the true in vivo pharmacological response of the human placenta itself remains poorly characterized. Moreover, with the advent of technologies like automated 3D imaging, machine learning-based morphometrics, microfluidic gradient systems, and bio-printing, the entire procedure is now faster and more precise (Figure 5) [60,61,62]. Downstream data analysis has advanced in parallel, incorporating automated 3D image segmentation and morphometric quantification, machine learning-based phenotypic classification, and statistical hit-calling frameworks that account for organoid- and patient-level variability in high-content screens [63,64].

### 6.3. Comparative Snapshot: Organoids and Other Models

Animal models have been heavily utilized in the field of reproductive toxicology but suffer from the lack of a species-specific and ethically sourced model. While primary human trophoblasts have more physiological relevance, they are severely limited by their availability, short longevity, and high variability [2]. There are new placenta-on-chip devices that integrate organoid-derived cells and microfluidics that mimic physiological flow and real-time monitoring capabilities [65,66]. That said, for HTS purposes, organoid trophoblasts are more easily scalable and analytically adaptable, and their potential future use in organ-on-chip devices is a promising synergistic solution [67,68].

### 6.4. Technical Challenges and Future Directions

Key challenges in trophoblast organoid work are that the efficiency of generation varies, an incomplete representation of the subtypes of trophoblast and limited incorporation of immune–vascular into the design [45]. The addition of maternal immune and endothelial cells may improve physiological relevance [69]. Standardized culture protocols, quality criteria and heterogeneous biobanks are mandatory for reproducibility and clinical applicability. In combination with HTS, AI, bioprinting and microfluidics or other support technologies, these stem-cell-derived trophoblast organoids will be essential as the main tools for precision obstetrics research into 2026 and further.

## 7. Benchmarking Placental Organoids: A Comprehensive Validation Landscape

Placental organoids recapitulate first-trimester trophoblast lineage composition, methylome profiles, and placental hormone secretion, representing molecularly and functionally relevant in vitro models [40,46,47]. In standard culture, however, these organoids adopt an inverted architecture—with syncytiotrophoblast facing inward rather than forming the outer surface as in vivo—so their physiological relevance should be understood at the molecular and functional level rather than as a faithful recapitulation of native villous architecture. They resemble in vivo trophoblast differentiation trajectories accurately when benchmarked on single cells, but they contain a very extensive progenitor-like compartment compared to their in vivo tissue counterpart [70]. Spatial and single-nucleus multiomics also reconcile organoid lineages to in situ trophoblast populations, and crucially resolve multinucleated syncytiotrophoblast, focusing interrogation of how faithfully organoids recapitulate the exchange surface and niche-specific signaling environments [71,72].

### 7.1. Single-Cell and Single-Nucleus Transcriptomics as the Gold Standard for Organoid Validation

Single-cell-resolution transcriptomics has become the central standard for validating trophoblast organoid fidelity. Shannon et al. systematically compared primary trophoblast-derived organoids (TBP-Org) and stem cell-derived organoids (TBS-Org) against first-trimester trophoblast, showing that both models reconstruct trophoblast differentiation trajectories, but TBP-Orgs more closely mirror in vivo global expression and cell-state heterogeneity [70]. They also found a larger progenitor-like population that is transcriptionally between villous CTB and EVT. This population is rare in vivo, which shows that the culture caused a change in the composition.

snRNA-seq has demonstrated its necessity for the precise characterization of syncytiotrophoblast (STB). Keenen et al. showed that conventional scRNA-seq recovers only 2.4–6% of STB in organoids, whereas snRNA-seq detects 38–76% of STB nuclei, closely matching stereological STB:CTB ratios (9:1 in tissue, 8:1 by snRNA-seq) [72]. Joint single-cell and single-nucleus profiling of placenta and organoids defined three conserved STB nuclear subtypes—juvenile (CTB/STB hybrid), oxygen-sensing (*FLT1*/hypoxia-enriched), and transport/GTPase signaling—demonstrating that organoids recapitulate syncytial nuclear heterogeneity.

Wang et al. advanced this standard by combining snRNA-seq with snATAC-seq in first-trimester and term placentas, as well as hTSC-derived organoids, delineating dynamic STB nuclear trajectories and identifying regulators such as *CEBPB*, *TFAP2A*, and *TFAP2C*, which were functionally validated in organoid-derived STB [67]. Karvas et al. showed that naïve hPSC-derived stem cell trophoblast organoids (SC-TOs) form five trophoblast clusters (CTB-1/2, STB-1/2, primitive EVT) in proportions and transcriptional states highly similar to primary organoids, with few differentially expressed genes, indicating that organoid culture represents a strong attractor state for trophoblast identity [73].

### 7.2. Epigenetic Profiling Reveals Chromatin-Level Fidelity and Culture-Dependent Modifications

Epigenetic benchmarking is now central to validating placental organoids, showing that they largely recapitulate primary placental methylation while revealing culture-specific features. Turco et al. created trophoblast organoids that clustered closely with first-trimester placentas on 850K methylation arrays (The Illumina Infinium MethylationEPIC (“850K”) array is a genome-wide platform that quantifies DNA methylation at ~850,000 CpG sites, enabling comparison of organoid methylomes against primary placental tissue). The correlations were strong, and the principal components were mostly made up of trophoblast markers like *CGB3*, *GATA3*, and *PSG6*, which clearly separated trophoblast samples from non-trophoblast samples [47]. Accurate trophoblast models must reflect trophoblast-specific methylation reprogramming, distinct from that of embryonic lineages [74]. Consistent with this, naive hPSC-derived (transdifferentiated) hTSCs acquire a broadly placenta-like methylome and largely sustain proper imprinted gene expression relative to placental hTSCs, with modest divergence confined to specific imprinted loci, suggesting partially compensatory regulation [75].

Functional epigenetic studies utilize organoids to elucidate chromatin regulators. Sadowski et al. utilized CUT&RUN to demonstrate that *BCOR* (*PRC1.1*) directly represses the stemness genes *CDX2* and *HAND1* during syncytiotrophoblast differentiation; the loss of *BCOR* had negligible effects in 2D but resulted in significant defects in 3D organoids, highlighting the significance of spatial context [76]. In the mouse, Tsolova et al. showed that age-related increases in H3K27ac/H3K4me3 at *Foxc1* in vivo are preserved in endometrial organoids derived from aged animals, which develop an epithelial hyperplasia phenotype—indicating that donor epigenetic memory is retained in culture. *Foxc1* overexpression reproduced similar transcriptomic and proliferative changes in human endometrial epithelial cells, but the aging phenotype itself, and its preservation in organoid culture, has not been established in human endometrial or trophoblast organoids and requires direct confirmation [77].

### 7.3. Proteomic Validation Demonstrates Functional Secretome Fidelity with Matrix-Dependent Variations

Proteomic benchmarking, while not as comprehensive as transcriptomics, has been essential for confirming the functional outputs of placental and endometrial organoids. Turco et al. used mass spectrometry on trophoblast organoid conditioned medium to find PSG1–9, GDF15, and hCGβ subunits. These were very similar to first-trimester villous secretomes, which showed that the proteins were made and released correctly [47].

Integrated miRNA-seq and secretome profiling of hormone-sensitive endometrial organoids revealed that sex steroids, through miR-3194-5p, regulate the basolateral secretion of AQP1, AQP9, and S100A9, increasing trophoblast migration and invasion by 50–80% in transwell assays [13]. Zhou et al. characterized apical intra-organoid fluid from fertile and infertile endometrial organoids, identifying 150 proteins (>1.5-fold dysregulated) whose altered secretion significantly compromised trophoblast spheroid adhesion (*p* < 0.0001), illustrating patient-specific dysfunctional proteomes with functional ramifications [78]. Matrix context further influences proteomes: a comparative analysis of trophoblast organoids in PEG versus Matrigel demonstrated syncytiotrophoblast-dominant and EVT-dominant phenotypes, respectively, highlighting the necessity to interpret proteomic benchmarks within the framework of culture conditions [79].

### 7.4. Morphological and Architectural Validation Confirms Villous-like Organization with Quantifiable Fidelity

Structural benchmarking demonstrates that trophoblast organoids autonomously organize into layered, villous-like architectures. In the standard Matrigel-embedded format, first-trimester trophoblast organoids develop a proliferative cytotrophoblast shell overlying an inner, multinucleated syncytiotrophoblast core—an inverted orientation relative to the villus in vivo, where syncytiotrophoblast forms the outer surface [80]. These organoids remain genetically stable and preserve this layered structure for over a year with regular passaging. Physiological, non-inverted orientation, with syncytiotrophoblast facing outward, has instead been achieved using suspension/agitation or air–liquid-interface culture formats. Haider et al. demonstrated that CTB-derived organoids autonomously produce STB and can be directed towards NOTCH1^+^ HLA-G^+^ EVT, maintaining the bipotential differentiation ability of primary cytotrophoblasts [81].

Next-generation hiPSC-derived placenta-like organoids integrate endothelial and stromal cells with CTB, STB, and EVT, achieving 90–99% trophoblast purity in sorted fractions and secreting hCG-β and VEGFA, while exhibiting appropriate responses to TNF-α and VEGFR inhibition, thus enhancing physiological relevance [82]. Sun et al. showed that organoid-forming capacity resides not in bulk differentiated cytotrophoblast but in a defined progenitor fraction termed the “side population” (SpTSCs)—cells identified by flow cytometry on the basis of their efflux of Hoechst 33342 dye via ABC transporters, a property historically used to enrich for stem-like cells. The resulting SpTSC-derived organoids (SpTSC-Org) mimic villous architecture but grow more slowly than organoids derived from Okae-protocol hTSCs [83].

### 7.5. Functional Validation Across Hormonal Secretion, Invasion, and Drug Transport

Endocrine, invasive, immune, and transport assays provide a comprehensive validation of placental organoids’ functionality. Trophoblast organoids produce physiologically relevant levels of hCG, progesterone, estrogens, PlGF, and placental lactogen, as measured by ELISA and mass spectrometry [47,72]. Culture conditions regulate gestational phenotype, with STBout organoids inducing late-pregnancy hormones CSH1, CSHL1, GH2, and STBin inducing early-pregnancy hormones, as measured by Keenen et al. [72]. Turco et al. demonstrated differentiation of trophoblast organoids into HLA-G+ EVT-like cells capable of invading Matrigel [47]; however, comprehensive functional validation of EVT identity—such as endovascular/spiral-artery mimicry or interstitial invasion in situ—was not established in this study and remains an open requirement for the field. scRNA-seq and trajectory analyses reveal a strong resemblance to in vivo EVT, with ligand-receptor predictions indicating appropriate interactions with dNK cells, as shown by Zhuang et al. [84]. The study of uNK-organoid co-culture shows that the cytokines made by uNK cells help control the later stages of EVT differentiation, including the processes related to blood flow and nutrient transport, as shown by Li et al. [85]. The barrier function of placenta organoids is validated through active drug transport, where trophoblast organoids show efflux activity for P-gp, BCRP, MRP1, and MRP2, with expression and localization similar to those of villous tissue, and commonly used antihypertensives and antipsychotics show transporter-specific inhibition patterns, as shown by Huang et al. [86].

### 7.6. Disease Modeling Demonstrates Clinical Relevance Through Preeclampsia and Infection Studies

Hypoxia-induced preeclampsia organoid cultures have also been developed, which recapitulated elevated sFLT-1/PlGF ratios, oxidative stress, and inhibition of the PI3K-AKT-mTOR pathway, while aspirin normalized sFLT-1/PlGF, restored PI3K-AKT-mTOR pathway activity, and corrected mitochondrial function [54]. In the companion L-NAME rat model of preeclampsia used in the same study, aspirin similarly upregulated CYP19A1 while inhibiting CYP1A1, corroborating the organoid-derived hormonal mechanism in vivo [54]. In addition, high-throughput screening of flame retardant EHDPP using trophoblast organoids identified disruption of normal villous structure, inhibition of IGF1R-dependent respiration, and impairment of invasion, while exposure to EHDPP in pregnant mice caused placental abnormalities, fetal growth restriction, failure of implantation, and stillbirth, validating translational toxicity prediction [87]. In addition, infectious disease modeling using human trophoblast stem cell-derived organoids showed high permissiveness to Zika virus (ZIKV) infection, mediated by expression of AXL, a TAM-family receptor tyrosine kinase, and TIM-1 (HAVCR1), a phosphatidylserine receptor—both known entry/attachment factors exploited by ZIKV and other enveloped viruses—providing a mechanistic basis for placental tropism and vertical transmission. Infected organoids mounted a limited interferon response and showed disruption of tissue structure, syncytialization, and hTSC stemness [51]. Similarly, stem cell-derived trophoblast organoids have also demonstrated biologically relevant susceptibility to SARS-CoV-2 infection, validating these systems as useful models of viral pathogenesis [73].

The stem-cell- and primary-CTB-derived trophoblast organoid cultures described above are increasingly being accompanied by transparent discussions of their limitations. Single-cell benchmarking has identified an expanded population of progenitor-like cells, intermediate in transcription between cytotrophoblast (CTB) and extravillous trophoblast (EVT), which are rare in primary tissue and likely reflect compositionally biased differentiation in culture [70]. Term-derived organoids often display transcriptional profiles associated with the first trimester and co-expression of both early (hCG) and late (chorionic somatomammotropin, CSH) hormones, suggesting organoids have converged on a default gestational state [72].

### 7.7. Systematic Identification of Limitations and Divergence from In Vivo Biology

Culture-specific artifacts are consistently observed, including enrichment in ribosome biogenesis, oxidative phosphorylation, and endoplasmic reticulum stress pathways, while extracellular matrix programs are underrepresented, consistent with the lack of a whole stromal niche environment. The process is further complicated by capture biases: standard droplet-based scRNA-seq protocols underrepresent large and multinucleated cells—including sizeable extravillous trophoblasts and multinucleated syncytiotrophoblasts—owing to cell-size and dissociation constraints, whereas snRNA-seq protocols sacrifice cytoplasmic RNA and underrepresent certain proliferative cell types. Integrated single-cell and single-nucleus RNA-sequencing protocols are currently the preferred choice for organoid RNA-sequencing analysis. The ratio of syncytiotrophoblast:cytotrophoblast in organoids is still closer to 1:1 compared with the ~9:1 predominance of syncytiotrophoblast observed in term placentas, even when using STBout or STBin culture conditions to manipulate polarity and differentiation.

The gestational stage: organoid stage matching is another challenge in the field: most protocols produce organoids with an early gestation-like phenotype regardless of gestational age at the time of sample collection, and ‘term’ organoids are smaller and have distinct transcriptional profiles, indicating intrinsic barriers to fully recapitulating late-gestation placental structure and function in vitro.

### 7.8. Multi-Omic Integration and Cross-Species Validation Strengthen Conclusions

Moving beyond descriptive profiling to establishing causal gene function requires integrating orthogonal data types with direct functional perturbation. Single-nucleus transcriptomic (snRNA-seq) and chromatin-accessibility (snATAC-seq) data can be integrated to nominate candidate transcriptional regulators of trophoblast identity, which are then tested directly by CRISPR perturbation in organoids; concordance between the predicted and observed phenotypes closes the loop between association and function [67]. Cross-species comparison must be interpreted with caution, because placental and trophoblast regulation diverge substantially between mouse and human; such comparisons are informative only for the minority of mechanisms that are demonstrably conserved, and are best used to generate hypotheses that are then tested directly in human cells. The shared BCOR loss-of-function phenotype between mouse placenta and human trophoblast stem cells is one such conserved example—but mouse data cannot substitute for human evidence when modeling human trophoblast [72,76], and murine trophoblast organoids have been shown to replicate in vivo knockout phenotypes for genes such as Nubpl and Gcm1 [88]. Patient-derived induced trophoblast stem cells from term cord tissue, which have been shown to share the transcriptomic identity with first-trimester trophoblast and have the ability to form multilineage organoids, have the potential for providing personalized disease models [29].

## 8. Future Directions, Research Gaps, and Emerging Technologies

### 8.1. Future Directions Emphasize Vascular Integration and Immune Complexity

The next generation of placental organoids will need to overcome several obvious shortcomings. For instance, while endothelial and vascular-like cell incorporation has been achieved, most placenta organoids lack a vascular network, and achieving this will likely require organoid integration with microfluidic placenta-on-chip systems [82]. Immune modeling in placenta organoids is incomplete, with co-culture with decidual NK cells having been achieved, while comprehensive immune modeling with macrophages, Tregs, and dendritic cells, with spatial interface atlases, remains to be achieved [71,84,85]. Another area that needs to be overcome is standardization, with considerable variability in conditions used to establish, passage, and differentiate placenta organoids, even with detailed protocols available, hindering reproducibility [80]. Fidelity scoring, illustrated by single-pipeline comparisons of placenta organoid platforms, will need to be achieved, with links to multi-omic, machine learning-derived composite indices to objectively benchmark and select placenta organoids [70].

### 8.2. Research Gaps in Trophoblast Lineage Specification and Organoid Biology

There are many aspects of trophoblast reprogramming and organoid systems that are not yet fully understood. The relationship between chromatin remodeling and transcription factor binding has not been clearly established. Live cell imaging has not clarified whether epigenetic modifications such as H3K27me3, H3K4me1/3, and 5mC/5hmC occur prior to or after transcription factor binding. The miRNA cluster C19MC, which is specific to primates, is a critical gate in trophoblast reprogramming. However, it is not clear whether this represents a rate-limiting step or merely one of several redundant pathways. Current methods for generating trophoblast organoids capture a limited range of developmental stages and yield a skewed cell-population composition, with single-cell benchmarking against primary tissue showing incomplete representation of CDX2-positive/negative stem cell populations, EVTs, STBs, and transitional cell states [70]. From a practical perspective, there are many challenges in establishing trophoblast organoids, including batch effects, a lack of quality control measures, culture artifacts such as expanded progenitors and inverted polarity, and difficulties in achieving reproducibility. Perhaps most importantly, placental organoids lack vasculature, maternal immune cells, and stroma. Thus, it has not been possible to model oxygen gradients, multicompartment immune tolerance, or maternal–fetal communication. Current disease modeling studies are descriptive in nature. Patient-derived organoids display abnormal networks. However, we are not yet able to use these systems for predictive purposes in patient prognosis or in informing therapeutic decisions.

Progress in this area is further constrained by jurisdiction-specific ethical and regulatory frameworks governing embryo- and placenta-derived material. Internationally, the ISSCR 2021 Guidelines govern stem cell- and embryo-model research; notably, they no longer treat the 14-day rule as an absolute ethical limit, instead recommending that research beyond 14 days may be considered on a case-by-case basis under rigorous scientific and ethical oversight. In the United Kingdom, the Human Fertilisation and Embryology Act and HFEA licensing govern the use of human embryos for research, including the derivation of embryonic stem cell and trophoblast stem cell lines; once derived, these cell lines are no longer regulated under the Human Fertilisation and Embryology Act, and their storage, distribution, and use are instead subject to other regulatory and governance frameworks, including deposition in the UK Stem Cell Bank where applicable. In the United States, the Dickey–Wicker Amendment restricts federal funding for embryo-destructive research, with additional variation in tissue-sourcing and consent requirements across states. These divergent frameworks constrain material availability, cross-institutional collaboration, and reproducibility in this field.

### 8.3. The Next Era

#### Trophoblast Organoids as Platforms for Modeling Placental Senescence: Convergence with Aging Biology, Dark Genome Reactivation, and Tauopathy

The advent of self-organizing trophoblast organoids that recapitulate villous architecture, cytotrophoblast-to-syncytiotrophoblast turnover (fusion-driven syncytial renewal), and extravillous invasive competence [47,81] offers a transformative platform for studying the premature cellular senescence that drives placental dysfunction and pregnancy failure [89]. Lessons from parallel aging-organoid disciplines provide both conceptual and methodological scaffolding: intestinal crypt organoids demonstrate that aged Lgr5^+^ stem cells exhibit impaired regenerative capacity reversible by mTOR inhibition [90]; choroid plexus and cerebral organoids derived from aged-donor iPSCs faithfully preserve neurodegenerative vulnerability [91]; and liver organoids from aged individuals accumulate somatic mutational burden mirroring clonal hepatocyte aging in vivo [92]. Crucially, iPSC reprogramming platforms have shown that epigenetic age—quantified by DNA methylation clocks—can be deliberately introduced or erased [93], enabling isogenic “aged” versus “rejuvenated” organoid pairs. Applied to trophoblast lineage reprogramming via GATA3/TFAP2C overexpression or BAP-driven naïve conversion [12], analogous strategies could generate trophoblast organoid systems differing primarily in epigenetic age, enabling precise senescence onset mapping.

A particularly compelling convergence emerges from the dark genome. De Cecco and colleagues from the Sedivy laboratory demonstrated that LINE-1 retrotransposons undergo progressive derepression during cellular aging, generating cytosolic reverse-transcribed DNA that activates cGAS-STING innate immune signaling and drives the interferon-rich senescence-associated secretory phenotype (SASP) [94]—a mechanism now being extended to organoid models of neurodegeneration and chronic inflammatory disease [95]. The placenta is one of the most LINE-1-permissive tissues in the human body, where retrotransposon dynamics contribute physiologically to ERV-driven syncytinization [96]; this very permissiveness renders trophoblasts acutely vulnerable to senescence-associated LINE-1 derepression cascades. Trophoblast organoids thus represent an ideal closed system to interrogate whether stress-induced premature senescence—triggered by hypoxia-reoxygenation, oxidative insult, or sFLT1-mediated ER stress—activates the LINE-1/cGAS-STING inflammatory axis [97], and whether retrotransposon suppression via nucleoside reverse transcriptase inhibitors or epigenetic stabilization of H3K9me3 can attenuate placental SASP and restore trophoblast functional integrity.

This dark genome framework acquires additional mechanistic depth when considered alongside an emerging and underappreciated dimension of placental pathology: tauopathy. Hyperphosphorylated tau, canonically associated with neurodegeneration, has emerged as a pathologically relevant mediator in non-neural tissues under proteotoxic and oxidative stress. Critically, Jash et al. demonstrated the presence of cis-phosphorylated tau (cis p-Tau)—the toxic, non-fibrillary conformer that evades PP2A-mediated dephosphorylation—in preeclamptic placentas, establishing tauopathy as a bona fide feature of placental pathology rather than a neurological epiphenomenon [98]. This conformational tau species promotes mitochondrial dysfunction, impairs proteostatic networks including the ubiquitin–proteasome and autophagy–lysosomal axes, and accelerates SASP activation in stressed trophoblasts—a convergence point with both replicative senescence and LINE-1-driven innate immune signaling. Trophoblast organoids engineered to overexpress cis p-Tau, or exposed to preeclampsia-relevant stressors, can now model how tau misfolding disrupts angiogenic signaling, syncytialization fidelity, and invasion competence in three-dimensional tissue context. Critically, integration of cis p-Tau perturbation with LINE-1 reporter systems and spatial proteomics will allow resolution of a key unresolved mechanistic question: whether tau-driven proteotoxicity operates upstream of retrotransposon derepression—by destabilizing heterochromatin through impaired nuclear lamina integrity—or downstream of cGAS-STING activation, as a consequence of chronic innate immune-driven translational stress. The answer has profound therapeutic implications, as it would position either tau conformational correctors, retrotransposon suppressors, or STING antagonists as rational entry points for attenuating premature placental senescence. Together, the convergence of trophoblast organoid technology with aging-organoid methodology, dark genome biology, iPSC epigenetic age engineering, and placental proteinopathy research positions this platform not merely as a model system, but as a discovery engine for mechanistically dissecting—and ultimately intercepting—the senescence cascades that underlie preeclampsia and placental insufficiency.

### 8.4. Precision Maternal–Fetal Interface Engineering and AI-Driven Therapeutic Discovery

The discipline is shifting towards dynamic and multi-scale approaches that incorporate mechanisms, phenotypes, and computational predictions. This shift is supported by four fundamental pillars. First, mechanistic precision involves live single-cell chromatin imaging in conjunction with transcriptomic, proteomic, and phospho-signaling analysis, and CRISPR disruption of C19MC and other key regulators to establish causal relationships and rationally optimize reprogramming efficiency. Second, physiologically integrated organoids that include perfused vasculature, decidual stroma, and spatially arranged maternal immune cells, according to spatial placental atlases, aim to recapitulate immune tolerance, oxygen sensing, and pathology. Third, standardization and scalability involve consensus protocols, artificial intelligence-based automated culture, machine learning-based quality control indices, and multi-site benchmarking to develop large and clinically annotated biobanks. Fourth, precision predictive medicine seeks to develop predictions of individual pregnancy outcomes and guide personalized prevention and therapy in obstetrics using organoid-derived multi-omic signatures and clinical data in advanced artificial intelligence models.

## Data Availability

The original contributions presented in this study are included in the article/. Further inquiries can be directed to the corresponding author.

## Abbreviations

The following abbreviations are used in this manuscript:

hTSCs	human trophoblast stem cells
iTSCs	induced trophoblast stem cells
hPSCs	human pluripotent stem cells
ESCs	embryonic stem cells
iPSCs	induced pluripotent stem cells
TE	trophectoderm
ICM	inner cell mass
STB	syncytiotrophoblast
EVT	extravillous trophoblast
hCG	human chorionic gonadotropin
